# Association between gingival bleeding and gingival enlargement and oral health-related quality of life (OHRQoL) of subjects under fixed orthodontic treatment: a cross-sectional study

**DOI:** 10.1186/1472-6831-12-53

**Published:** 2012-11-27

**Authors:** Fabricio Batistin Zanatta, Thiago Machado Ardenghi, Raquel Pippi Antoniazzi, Tatiana Militz Perrone Pinto, Cassiano Kuchenbecker Rösing

**Affiliations:** 1 Department of Stomatology, Universidade Federal de Santa Maria (UFSM), Rua Tiradentes, 76/801, Centro, Santa Maria, Zip Code 97050-730, RS, Brazil; 2 Centro Universitário Franciscano (UNIFRA), Santa Maria, RS, Brazil; 3 Universidade Federal do Rio Grande do Sul (UFRGS), Porto Alegre, RS, Brazil

**Keywords:** Quality of life, Epidemiology, Risk factors, Orthodontics, Gingivitis, Gingival hyperplasia

## Abstract

**Background:**

There are scarce evidences that evaluated the impact of periodontal disease on oral health-related quality of life (OHRQoL) taking marginal gingival alterations into consideration. Thus, this study aimed to verify the association between OHRQoL and gingival enlargement and gingival bleeding in subjects under fixed orthodontic treatment (FOT).

**Methods:**

330 participants under FOT for at least 6 months were examined by a single, calibrated examiner for periodontal variables and dental aesthetic index. Socio-economic background, body mass index, time with orthodontic appliances, and use of dental floss were assessed by oral interviews. OHRQoL was evaluated using the oral health impact profile (OHIP-14) questionnaire. The assessment of associations used unadjusted and adjusted Poisson regression models.

**Results:**

Higher impacts on the OHIP-14 overall were observed in subjects who presented higher levels of anterior gingival enlargement (RR 2.83; 95% CI 2.60-3.09), were non-whites (RR 1.29; 95% CI 1.15-1.45), had household income lower than five national minimum wages (RR 1.85; 95% CI 1.30-2.61), presented body mass index > 25 (RR 1.14; 95% CI 1.01-1.29), and showed a dental aesthetic index > 30 (RR 1.32; 95% CI 1.20-1.46).

**Conclusions:**

Anterior gingival enlargement seems to influence the OHRQoL in subjects receiving orthodontic treatment.

## Background

Quality of life (QoL) is conceived as an eminently human notion that is a reflection of the degree of satisfaction with one’s family and social life [1]. It presupposes the ability to perform a cultural synthesis of many aspects of life that reflect the knowledge, experience and values of individuals and groups over the ages; thus, it is a social construct having the cultural relativity of social elements in its model of comfort and well-being [1].

Oral health-related quality of life (OHRQoL) is defined by self-reports specifically pertaining to oral health, capturing both the functional, social and psychological impacts of oral disease [2]. Oral epidemiology has used indices to assess the self-reporting of OHRQoL issues as adjuncts to clinical examinations, thereby documenting the full impact of oral disorders. There is a range of instruments designed for these aims [3]. The OHIP_14_ is one of these instruments; it has been widely used in several cross-sectional and longitudinal studies including adolescents [4] and young adult [5] populations under orthodontic treatment. It is divided in to seven subscales, grouping functional limitation, physical pain, psychological discomfort, physical and psychological disabilities and handicap [3]. The questionnaire was developed in Australia [6], but further studies confirmed its validity and reliability in Brazilian population [7].

Periodontal disease is usually documented by therapist-centered surrogate endpoints such as bleeding on probing (BOP), probing depth PD and clinical attachment level (CAL). However, patient-centered measures have recently begun to be explored [1]. These methods have attempted to measure the impact of periodontal status on patients’ lives, and have shown that periodontal disease does have an impact on patients’ quality of life [8-13] and recently it has been demonstrated by a systematic review that periodontal treatment can moderately improve OHRQoL [14]. Thus, the identification of this impact may provide an opportunity for promoting more effective and suitable actions to be employed as complements to normative criteria [15].

Clinical studies have indicated that orthodontic treatment may be associated with deterioration in periodontal health [16-18]. However, the majority of studies have concluded that overall gingival alterations are transient with no permanent damage to periodontal supporting tissues [19-22].

To the best of our knowledge, there are no studies evaluating the OHRQoL impact of periodontal conditions on individuals receiving orthodontic therapy. This study aimed to evaluate the independent associations between OHRQoL and gingival enlargement and gingival bleeding.

## Methods

This cross-sectional survey examined a group of subjects, 14 to 30 years of age, who were receiving orthodontic treatment in an orthodontic graduate clinic in Santa Maria, a city in southern Brazil. The study protocol was reviewed and approved by the Committee of Ethics in Research of the Franciscan University Center (protocol n^o^ 063.2010.2). Subjects who agreed to participate signed an informed consent form. Patients diagnosed with oral pathological conditions (i.e. gingivitis, periodontitis, active caries, semiological lesions, …) were advised to seek consultation and treatment.

### Eligibility criteria

To be eligible for the study, individuals must have been under fixed orthodontic treatment for at least six months. Exclusion criteria comprised presence of diseases and conditions that could pose health risks to the participant or that could interfere with the clinical examination. Individuals who had undergone antibiotic therapy within three months prior the examination were also excluded. Female subjects were excluded if they were pregnant or breastfeeding. Individuals were excluded if they required a prophylactic regimen of antibiotics for clinical examination.

### Study sample

The orthodontic dental clinic of the UNINGÁ-SM has been treating during the data collection period an estimated population of seven hundred patients. Of these, approximately six hundred individuals presented for continuation of treatment and were assessed for eligibility criteria. This resulted in four hundred eligible individuals according eligibility criteria. Of these, 330 were evaluated, resulting in a non-response rate of less than 20%. The main reason of non-response subjects was due to abandonment of orthodontic treatment. In the present investigation, the study sample comprised 330 individuals aged 14 to 30 years, 171 (51.8%) female and 159 (48.2%) male, 263 (79.7%) white and 67 (20.3%) non-white. The clinical examinations were performed between September/2009 and December/2010. In general, the orthodontic treatment consisted of fixed straight-wire appliances used in all subjects, the first treatment phase consisted in alignment and leveling of the teeth with NiTi and stainless steel archwires; following, the space closure was proceeded by sliding mechanics with a 0.019x0.025“stainless steel archwires and elastomeric ligatures; and last, in the finalization phase 0.018” stainless steel archwires was used to promote adequate intercuspidation.

### Examinations

A single, calibrated examiner (FBZ) performed all clinical examinations, with the aid of one dental assistant for recording. All permanent, fully erupted teeth, excluding third molars, were examined using a manual periodontal probe (Neumar®, São Paulo, SP, Brazil). Six sites (mesio-buccal, mid-buccal, disto-buccal, disto-lingual, mid-lingual, and mesio-lingual) were assessed for each tooth.

Teeth in each quadrant were dried with a blast of air, and plaque index (PlI) [23] and gingival index (GI) [24] indexes were recorded. Probing depth (PD) (distance from the free gingival margin to the bottom of the pocket/sulcus), attachment loss (distance from the cement-enamel junction (CEJ) to the bottom of the pocket/sulcus) and bleeding on probing (BOP) were then assessed. However, due most participants in this study presented low mean values for PD and CAL, these data were therefore not used in the present analysis. Next, gingival enlargement [25] was evaluated. The degree of gingival thickening in both labial and lingual aspects was graded as follows: 0 = normal; 1 = thickening from the normal up to 2 mm; 2 = thickening from normal greater than 2 mm. The extent of encroachment of the gingival tissues onto the adjacent crowns was also graded, using 0 (normal), 1 (up to 1 mm in occlusal/medial direction), 2 (up to 2 mm in occlusal/medial direction) and 3 (≥ to 3 mm in occlusal/medial direction), on the labial and lingual surfaces. The 2 scores (thickening and gingival encroachment) were added, thus giving a hyperplasia score for each gingival unit. The maximum score obtainable using this method is 5. Additionally, the excess resin was dichotomously assessed by inspection with a probe around the bracket on the buccal surfaces of each bonded bracket. The buccal surface was divided into distal, mesial and cervical aspects. Excess resin, less than 1 mm distance from the gingival margin, was considered present.

After clinical examination, socioeconomic and demographic data were collected using a structured, written questionnaire. Race was scored as white or non-white. Household income information was measured in terms of the Brazilian minimum wage, which corresponded to approximately US$290 (US dollars) during the period of data gathering. The income information was measured dichotomously (up to 5 national minimum wages/> 5 national minimum wages). Pll and GI were dichotomized as visible plaque (present/absent) and gingival bleeding (present/absent), respectively with scores 0 and 1 of each index being considered “absent” and scores 2 and 3 being considered “present”. The percentages of sites per person with visible plaque, gingival bleeding and bleeding on probing were calculated. The questionnaire also reported the declared frequency of use of dental floss. Regular interdental hygiene was defined as regular use of dental floss at least once a day. Non-users of dental-floss were defined as subjects who did not use interdental oral hygiene devices every day or who did not perform interdental hygiene. Body mass index was also calculated by dividing weight by (height). Malocclusion was evaluated by a dental aesthetic index (DAI) [26]. According to the overall DAI scores, subjects were classified as minor malocclusion (<30 DAI score) or severe malocclusion (≥ 30 DAI score).

At the end of each interview, a subjective measurement tool for the impact of oral health on quality of life was applied. This assessment tool was the OHIP-14 [6]. This instrument is a questionnaire containing fourteen questions addressing the following dimensions, based on the theoretical and conceptual model of oral health [27]: Functional limitation, physical pain, psychological discomfort, physical disability, social disability and handicap. Each of the seven subscales has two questions graded on a five-point scale, for which patients choose an answer using the following codes: 0 = never; 1 = hardly ever; 2 = occasionally; 3 = fairly often; 4 = very often. OHIP-14 total scores were calculated using an additive method. A single researcher conducted all interviews.

### Measurement reproducibility

The examiner was trained and calibrated in performing the clinical measurements, before doing the examinations. Gingival enlargement index [25], was described to be assessed by upper and lower full mouth alginate impressions and a study model. Due to the difficulty of doing study models for all patients, gingival enlargement was assessed in an intra-oral clinical examination. To assure the fidelity of GE between clinical examination and study model assessment, both measures were compared using Intraclass Correlation Coefficient (ICC) for 5 subjects, with generalized gingival hyperplasia in at least 3 sites, resulting in an ICC of 0.92. After that, GE reproducibility was assessed using ICC at the site level in 15 subjects, resulting in an ICC of 0.86.

### Data analysis

For this exploratory analysis, data pattern distribution was analyzed and non-parametric (Mann–Whitney, Kruskal-Wallis and Spearman correlation) tests were used. After a descriptive analysis of the overall scores of OHIP-14 was done, the overall mean of OHIP-14 scores and the means of the individual domains were then analyzed for differences among demographic characteristics, socioeconomic indicators and clinical status. Unadjusted Poisson Regression analysis with robust variance was performed to correlate the overall mean OHIP-14 score with each demographic, socioeconomic and clinical indicator. In this analysis, the outcome was considered as a continuous outcome and rate ratios, which correspond to the quotient between average scores of each comparison group and 95% confidence intervals (95% CI), were calculated. A multivariate model was later run with the covariates. These covariates were selected using a backward stepwise procedure. To be entered into the model, only variables with p ≤ 0.20 were used; in order to be retained in the final multivariate model, the variables should present p ≤ 0.05. The statistical software STATA 9.0 (Stata Corp, College Station, USA) was used for all analyses.

## Results

All subjects from the cross-sectional survey completed the questionnaire and clinical examination. Considering the periodontal diagnosis, based on patient ages and periodontal data explored in another study [28] (2,06 mm ± 0,18 and 1,6 ± 0,11 for mean probing depth and clinical attachment levels in proximal sites, respectively) most patients have only periodontal diagnosis of gingivitis. The demographic characteristics and socioeconomic and clinical status of the subjects are shown in first column of Table 1. Subjects were predominantly white; almost 50% were older than 19 years. The majority of individuals presented monthly average income less than 5 minimum wages. Almost 90% of subjects had a BMI lower than 25, and about one third (27.7%) presented severe malocclusion (DAI >30). A total of 145 (43.9%) subjects had been receiving orthodontic treatment for more than one year, and 81 (24.5%) subjects used dental floss every day. The mean value for anterior gingival enlargement and percentage of whole mouth gingival bleeding (percentage mean of gingival marginal gingival bleeding) were 0.69 and 58.72%, respectively.

**Table 1:** The demographic characteristics and clinical status of the subjects

**Variables**	**n (%)**
***Gender***	
Male	159 (48.1)
Female	171 (51.8)
**Ethnicity**	
White	263 (79.7)
Non-White	67 (20.3)
**Age (years)**	
14-19	162 (49.0)
20-24	121 (36.6)
25-30	47 (14.2)
**Household Income**	
≤ 5 wages	270 (81.8)
> 5 wages	60 (18.1)
***BMI (Kg/m***^***2***^***)***	
<25	296 (89.7)
≥ 25	34 (10.3)
**TUFOT (Months)**	
6-12	263 (79.7)
> 12	67 (20.3)
***DAI***	
***<30***	*237 (71.8)*
***≥30***	*93 (27.7)*
***Dental floss***	
Users	81 (24.5)
Non-users	249 (75.5)

OHIP-14 scores had an anormal distribution with scores ranged from 0 to 8 and presented an average of 2.74 (SD = 2.1). Domain-specific scores presented small variation, and psychological discomfort was the domain with the largest variation (0–4). Social disability and handicap presented “never” scores for all participants (Table 2).

**Table 2:** Descriptive distribution of overall and domain-specific OHIP-14 scores

	**Number of items**	**Mean OHIP-14**	**Median OHIP-14 scores**	**Possible range**	**Observed range**
		**scores (±SD)**	**(P25 –P75)**		
**OHIP-14 (*overall scale*)**	14	2.74 (±2.10)	2 (0–8)	0-56	0-8
**Domains**					
Functional limitation	2	0.21 (±0.41)	0 (0–1)	0-8	0-1
Physical pain	2	0.25 (±0.63)	0 (0–2)	0-8	0-2
Psychological discomfort	2	1.95 (±0.93)	2 (0–2)	0-8	0-4
Physical disability	2	0.51 (±0.21)	0 (0–1)	0-8	0-1
Psychological disability	2	0.26 (±0.78)	0 (0 – 0	0-8	0-3
Social disability	2	0 (0)	0 (0)	0-8	0
Handicap	2	0 (0)	0 (0)	0-8	0

Table 3 shows the OHIP-14 dichotomization in two categories: (1) scores of “never”, “hardly ever” and “occasionally”; and (2) scores of “fairly often” and “very often”. Most domains did not present an important impact, with the majority of those answers in category 1. Psychological discomfort was the domain that had the largest percentage of responses in category 2, with 17.2% of the answers (Table 3).

**Table 3:** Frequency of answers with important and without important impact of OHIP-14 domains

**OHIP-14 questions**	**Domains**	**“Never”, “hardly ever” or “occasionally”**	**“Very often” or “fairly often**
		**n (%)**	**n (%)**
1. Had trouble pronouncing any words	Functional limitation (1,2)	291 (88.1)	39 (11.8)
2. Felt sense of taste has worsened			
3. Had painful aching	Physical pain (3,4)	330 (100)	0 (0)
4. Found it uncomfortable to eat any foods			
5. Been self-conscious		273 (82.7)	57 (17.2)
6. Felt tense			
7. Diet has been unsatisfactory	Physical disability (7,8)	330 (100)	0 (0)
8. Had to interrupt meals			
9. Found it difficult to relax	Psychological disability (9,10)	313 (94.8)	17 (5.1)
10. Been a bit embarrassed			
11. Been a bit irritable	Social disability (11,12)	330 (100)	0 (0)
12. Had difficulty doing usual jobs			
13. Felt life less satisfying	Handicap (13,14)	313 (94.8)	17 (5.1)
14. Been totally unable to function			
OHIP-14 (overall scale)		273 (82.7)	57 (17.2)

The Unadjusted Poisson Regression analysis, performed for domain-specific OHIP-14 scores, demonstrated statistical associations between functional limitation, psychological discomfort and physical disability for almost all covariates. Physical pain was associated with age, household income, time receiving orthodontic treatment, dental aesthetic index and anterior gingival enlargement. Psychological disability demonstrated association with subjects’ education, body mass index, time receiving orthodontic treatment, dental aesthetic index and anterior gingival enlargement. On the other hand, social disability and handicap were not associated with any of the covariates (Table 4).

**Table 4:** Univariate analysis between socioeconomic factors and oral clinical conditions related to domain-specific OHIP-14 scores

**Variables**	**n (%)**	**Fl**	**Php**	**Psydisc**	**Phydisa**	**Psydisa**	**Sdisa**	**Hand**
					**Mean (±SD)**			
***Gender***		p* < 0.001	p*0.926	p*0.033	p* < 0.001	p*0.385	p* > 0.999	p* > 0.999
Male	159 (48.1)	0.44 (0.49)	0.21 (0.53)	1.81 (1.21)	0.10 (0.30)	0.22 (0.71)	0 (0)	0 (0)
Female	171 (51.8)	0 (0)	0.29 (0.70)	2.07 (0.53)	0 (0)	0.30 (0.84)	0 (0)	0 (0)
**Ethnicity**		p* < 0.001	p*0.728	p* < 0.001	p* < 0.001	p*0.124	p* > 0.999	p* > 0.999
White	263 (79.7)	0.04 (0.21)	0.26 (0.66)	1.83 (0.94)	0.02 (0.16)	0.30 (0.84)	0 (0)	0 (0)
Non-White	67 (20.3)	0.86 (0.34)	0.20 (0.47)	2.4 (0.73)	0.14 (0.35)	0.11 (0.47)	0 (0)	0 (0)
**Age (years)**		p** < 0.001	p** < 0.001	p** < 0.001	p** < 0.001	p**0.052	p** > 0.999	p** > 0.999
14-19	162 (49.0)	0.11 (0.32)	0.30 (0.72)	2.16 (0.46)	0 (0)	0.36 (0.91)	0 (0)	0 (0)
20-24	121 (36.6)	0.27 (0.44)	0.13 (0.49)	1.52 (0.97)	0 (0)	0.23 (0.71)	0 (0)	0 (0)
25-30	47 (14.2)	0.40 (0.49)	0.38 (0.53)	2.29 (1.50)	0.36 (0.48)	0.04 (0.29)	0 (0)	0 (0)
**Household Income**		p*0.468	p* < 0.001	p* < 0.001	p*0.046	p* 0.966	p* > 0.999	p* > 0.999
≤ 5 wages	270 (81.8)	0.20 (0.40)	0.31 (0.68)	2.27 (0.57)	0.06 (0.24)	0.27 (0.81)	0 (0)	0 (0)
> 5 wages	60 (18.1)	0.25 (0.43)	0 (0)	0.50 (0.87)	0 (0)	0.23 (0.64)	0 (0)	0 (0)
***BMI *(*Kg*/*m***^***2***^**)**		p* < 0.001	p*0.520	p* < 0.001	p* < 0.001	p* 0.032	p* > 0.999	p* > 0.999
<25	296 (89.7)	0.14 (0.35)	0.25 (0.64)	1.88 (0.92)	0.03 (0.18)	0.30 (0.82)	0 (0)	0 (0)
≥ 25	34 (10.3)	0.82 (0.38)	0.23 (0.49)	2.55 (0.82)	0.20 (0.41)	0 (0)	0 (0)	0 (0)
**TUFOT (Months)**		p*0.027	p* < 0.001	p*0.154	p* < 0.001	p* < 0.001	p* > 0.999	p* > 0.999
6-12	263 (79.7)	0.25 (0.43)	0.03 (0.25)	1.95 (0.52)	0 (0)	0.05 (0.32)	0 (0)	0 (0)
> 12	67 (20.3)	0.15 (0.36)	0.53 (0.82)	1.95 (1.28)	0.11 (0.32)	0.54 (1.07)	0 (0)	0 (0)
***DAI***		p* < 0.001	p* < 0.001	p* < 0.001	p* < 0.001	p* < 0.001	p* > 0.999	p* > 0.999
***<30***	*237* (*71*.*8*)	0.07 (0.25)	0.10 (0.37)	1.87 (0.94)	0.07 (0.25)	0 (0)	0 (0)	0 (0)
***≥30***	*93* (*27*.*7*)	0.58 (0.49)	0.62 (0.93)	2.15 (0.88)	0 (0)	0.95 (1.25)	0 (0)	0 (0)
***Dental floss***		p* < 0.001	p*0.138	p* < 0.001	p* < 0.001	p*0.094	p* > 0.999	p* > 0.999
Users	81 (24.5)	0.39 (0.49)	0.24 (0.48)	1.50 (1.56)	0.20 (0.40)	0.12 (0.48)	0 (0)	0 (0)
Non-users	249 (75.5)	0.15 (0.36)	0.25 (0.67)	2.09 (0.53)	0 (0)	0.31 (0.86)	0 (0)	0 (0)
**AGE**								
	330 (100 %)	p***0.003	p*** < 0.001	p***0.005	p***0.095	p***0.00	-	-
**WMGB**								
	330 (100 %)	p***0.134	p***0.065	p***0.005	p*** < 0.001	p***0.71	-	-

FL functional limitation; Php physical pain; Psydisc psychological discomfort; Phydisa physical disability; Psydisa psychological disability; Sdisa social disability; Hand handicap; AGE anterior gingival enlargement; BMI body mass index; TUFOT time under fixed orthodontic treatment; DAI dental aesthetic index; WMGB whole mouth gingival bleeding.

p* = Mann Whitney test; p** = Kruskall Wallis test; p*** = Spearman Correlation.

The unadjusted Poisson Regression assessment of associations revealed race (RR 1.50; 95% CI 1.30-1.74), household income (RR 3.18; 95% CI 2.09-4.84), body mass index (RR 1.46; 95% CI 1.22-1.74), time receiving orthodontic treatment (RR 1.50; 95% CI 1.30-1.74), dental aesthetic index (RR 2.02; 95% CI 1.74-2.35), and anterior gingival enlargement (RR 3.55; 95% CI 3.29-3.82) as the main covariates of the overall OHIP-14 score. The multivariate regression model also associated ethnicity (RR 1.29; 95% CI 1.15-1.45), with non-white subjects reporting lower overall OHIP-14 scores; household income (RR 1.85; 95% CI 1.30-2.61), with subjects whose family income was less than five national minimum wages reporting lower overall OHIP-14scores; body mass index (RR 1.14; 95% CI 1.01-1.29), with subjects who presented a BMI > 25 reporting lower overall OHIP-14 scores; and dental aesthetic index (RR 1.32; 95% CI 1.20-1.46), with subjects who showed DAI > 30 reporting lower overall OHIP-14 scores. Higher levels of anterior gingival enlargement were associated with a 2.83 fold increase in overall OHIP-14 scores (RR 2.83; 95% CI 2.60-3.09 (Table 5).

**Table 5:** Unadjusted (RR) and Adjusted (RR*) assessments between independent variables associated to overall OHIP-14 scores

**Variables**	**n (%)**	**OHIP-14 Mean (SD)**	**OHIP-14 RR (95% CI)**	OHIP-14 RR* (95% CI)
***Gender***			**p 0.567**	**
Male	159 (48.1)	2.67 (±1.89)	1.00	
Female	171 (51.8)	2.84 (±2.31)	0.95 (0.80 – 1.12)	
***Ethnicity***			**p < 0.001**	**p < 0.001**
White	263 (79.7)	2.48 (±2.13)	1	1
Non-White	67 (20.3)	3.74 (±1.62)	1.50 (1.30 -1.74)	1.29 (1.15 – 1.45)
**Age (years)**			**p < 0.001**	**
14-19	162 (49.0)	2.16 (±1.81)	0.62 (0.47 -0.81)	
20-24	121 (36.6)	2.95 (±1.96)	0.84 (0.65 -1.0)	
25-30	47 (14.2)	3.48 (±2.83)	1	
**Household Income (Wages)**			**p < 0.001**	**p < 0.001**
≤ 5	270 (81.8)	3.13 (±2.0)	3.18 (2.09 – 4.84)	1.85 (1.30 – 2.61)
> 5	60 (18.1)	0.98 (±1.61)	1	1
***BMI *(*Kg*/*m***^***2***^**)**			**p < 0.001**	**p 0.033**
<25	296 (89.7)	2.61 (±2.10)	1	1
≥ 25	34 (10.3)	3.82 (±1.78)	1.46 (1.22 -1.74)	1.14 (1.01 – 1.29)
**TUFOT (Months)**			**p < 0.001**	**
≤ 6-12	263 (79.7)	2.29 (±0.76)	1	
> 12	67 (20.3)	3.31 (±2.96)	1.50 (1.30 – 1.74)	
***DAI***			**p < 0.001**	**p < 0.001**
***<30***	237 (71.8)	2.12 (±1.58)	1	1
***≥30***	93 (27.7)	4.31 (±2.43)	2.02 (1.74 – 2.35)	1.32 (1.20 -1.46)
***Dental floss***			**p 0.315**	
Users	81 (24.5)	2.48 (±2.76)	1	
Non-users	249 (75.5)	2.82 (±1.83)	1.13 (0.88 – 1.46)	
**AGE**			**p < 0.001**	**p < 0.001**
	330 (100%)	__	3.55 (3.29 – 3.82)	2.83 (2.60 -3.09)
**WMGB**			**p 0.790**	
	330 (100%)	**__**	1.00 (0.99 – 1.00)	

RR rate ratio; AGE Anterior gingival enlargement; BMI body mass index; TUFOT time under orthodontic fixed appliance; DAI dental aesthetic index; WMGB whole mouth gingival bleeding.

*Adjusted by gender, ethnicity, age, Household Income, BMI, TUFOT, DAI, dental floss use, AGE and WMGB.

** Variables not included in the final multiple model after adjustment RR rate ratio; AGE Anterior gingival enlargement; BMI body mass index; TUFOT time under orthodontic fixed appliance; DAI dental aesthetic index; WMGB whole.

## Discussion

The present study demonstrated that gingival enlargement is associated with OHRQoL. Regarding factors associated with the impact of oral conditions on OHRQoL, the most frequently reported variables in the literature were caries and socioeconomic status [29]. When the impact of periodontal disease on OHRQoL is evaluated, clinical attachment loss or probing pocket depth have been used to verify this association, but without taking marginal gingival alterations into consideration [12,13,30]. In the present study, anterior gingival enlargement was independently associated with overall OHIP-14 scores.

In the last years, the OHIP-14 instrument has been used to verify associations between OHRQoL and periodontal conditions. Our study revealed a relatively small variation among OHIP-14 scores. On the other hand, previous studies presented larger variation. Araújo et al. ^2^ demonstrated a variation in overall OHIP-14 scores, ranging between 36–56 points in 61.2% of subjects with periodontal disease, defining CAL as the endpoint. Meanwhile, our study found a maximum value of 8 points. Ng and Leung^7^ demonstrated that subjects from a Chinese population, with full-mouth mean CAL above 3 mm, scored significantly higher on the impact of oral health on their OHRQoL in the overall OHIP-14and several subscales. Similar results were found by Bernabé and Marcenes^11^, who considered individuals to have periodontal disease to be those having at least two proximal sites with CAL ≥ 4 mm, and at least one proximal site with PD ≥ 4 mm. Differences related to the criteria for defining periodontal disease may explain the differences with the results from our study, related to the level of impact of periodontal disease on OHRQoL. In the present study, we did not use PD or CAL as independent variables due to the low mean values of PD and CAL (2.06 mm and 1.60 mm, respectively). We therefore used anterior gingival enlargement to report periodontal alterations, in order to evaluate whether a gingival aesthetic aspect would impact patients’ OHRQoL. Moreover, to the best of our knowledge, evidence evaluating the impact of gingival enlargement associated with orthodontic treatment on OHRQoL is nonexistent. From this perspective, the present study gives new information.

Gingival appearance is one of the components of the harmony of the smile, which is seen as one of the most important physical characteristics of the development of self-image and self-esteem. It expresses feelings of joy, success, sensuality, affection, and reveals self-confidence and kindness. People who are satisfied with their smile appear to be more self-confident and have higher self-esteem than those who are dissatisfied [31]. Subjects whose dental and gingival aspects are not in agreement with social patterns are most commonly affected with an unpleasant appearance [31-33]. Evidence shows that gingival enlargement (GE) is associated with aesthetic impairment and, in more severe cases, with phonetic alterations and masticatory problems [34,35]. Thus, higher levels of age may show increased emotional and social problems, which could be reflected in the association with higher means of overall OHIP-14. GE associated with orthodontic treatment is a hypertrophic form of gingivitis. The exact mechanism for the development of GE is not completely understood, but probably involves increased production by fibroblasts of amorphous ground substances with a high level of glycosaminoglycans. Increases in the mRNA expression of type I collagen and up-regulation of the keratinocyte growth factor receptor could play important role in the excessive proliferation of epithelial cells and the development of GE [36]. In some studies, poor oral hygiene enhanced GE [37,38], while other clinical studies concluded that overall gingival changes during orthodontic treatment are transient with no permanent damage to the periodontal supporting tissues [19,22]. Another hypothesis that may partly explain our results is the age range of the subjects, which ranged from 14 to 30 years. In this age group, aesthetic appearance seems to be more valued than by those of younger or older ages [39].

According to the dichotomization of “important impact” (scores of “fairly often” or “very often” in one or more of the OHIP-14 items) of oral health status on OHRQoL, 17.2% of subjects reported important impact in the psychological discomfort domain (been self-conscious and/or felt tense), while 11.8% and 5.1% reported important impact in the functional limitation and psychological disability domains (found difficult to relax and/or felt embarrassed), respectively. The analysis of the psychological discomfort and psychological disability domains revealed that subjects with higher means of anterior gingival enlargement had a greater level of concern and embarrassment than did subjects with lower means of anterior gingival enlargement. However, we found that most of the responses to the OHIP-14 domains showed little impact (“never”, “hardly ever” or “occasionally”) of oral conditions on OHRQoL. Another study demonstrated that the most frequent responses to OHIP-14 were “fairly often” and “very often”, for both the relaxation dimension and the embarrassment felt by patients with periodontal disease [8]. It has to be highlighted that subjects participating in that study had clinical diagnosis of periodontitis, while most subjects of our study had only gingivitis. It is to be expected to find a higher mean impact on OHRQoL in subjects with periodontitis because it is associated with a wide range of clinical signs and symptoms such as bleeding, tooth mobility, receding gums, bad breadth and toothache, which may have considerable impact on the daily life of subjects with more severe periodontal disease [10,13,30].

Variables related to socio-demographic characteristics, like ethnicity and household income, were demonstrated to influence subjects’ perception of OHRQoL. It has been established that individuals from different socioeconomic backgrounds are exposed to different risk factors that affect oral health and, consequently, perceive the impact of oral health on OHRQoL in different ways [40]. Moreover, individuals with lower socioeconomic status are subjected to material deprivation which could influence their engaging in riskier behaviors, resulting in more severe impacts on their OHRQoL [41].

The present study did not find differences on overall OHIP-14 scores, after adjustment, between the genders. This result disagrees with a previous study that reported higher impact of oral health on OHRQoL in women as compared to men [42]. This result could be explained by one of the following hypotheses: first, in subjects with orthodontic appliances, females present a similar concern with aesthetic-related issues than men. Second, the lack of extreme differences related to socioeconomic status in our sample may have influenced the results, since self-reported health-related quality-of-life between males and females may be influenced by sociodemographic and socioeconomic status [40]. Third, our study may have lacked adequate power to detect differences between men and women.

The results of the present study corroborate several studies demonstrating that socioeconomic gradients affect the prevalence of oral health problems [41]. Non-white subjects present a higher gradient of oral disease, and this fact cannot be theoretically related to socioeconomic disparities. Thus, compared to their white counterparts, non-whites have a greater likelihood of perceiving a higher impact of oral health on their OHRQoL [43].

Our findings showed that severe malocclusion was found to be associated with higher overall OHIP-14 scores. DAI was used to measure the degree of malocclusion and it’s aorthodontic index based on socially defined aesthetic standards. It is useful in epidemiological surveys to identify the need for orthodontic treatment, and also as a screening device to determine priority for subsidized orthodontic treatment. Cut-off points for the determination of severe malocclusion were previously reported [26]. Patients with severe malocclusions may report various oral health impacts that affect their well-being in many ways [44].

Data analysis showed that excess body weight is associated with OHRQoL. One interpretation of these results is that there is a body standard for attractiveness in a larger cultural context. Accordingly, subjects constantly confronted with the media’s slender and beautiful renderings may aspire to an ideal quite impossible for most of them to attain. Not achieving these ideals, they end up with low self-esteem [40].

It’s well established that socioeconomic status and other dental clinical variables have a negative impact on OHRQoL [3,45]. Therefore, the associations between gingival bleeding and gingival enlargement with OHRQoL were assessed taking into account the possible confounding effects of these variables. Studies assessing the association between gingival enlargement and its impact in OHRQoL in orthodontic subjects, using multiple regression analyses controlled for other sociodemographic and clinical variables which may act as confounders, are nonexistent. In addition, Poisson regression with robust variance was used in order to provide PR estimates, which are easier to interpret than odds ratios. This study had a cross-sectional design, which hypothesizes relations between the outcome and predictor variables, without establishing causal relationships. This is a limitation of this study. However, conclusions from cross-sectional studies are important for identifying indicators to be included in longitudinal or even, experimental studies [46]. The present study involved 330 orthodontic patients attending a private orthodontic specialist training program. This convenience sample limited the extent to which these findings can be generalized to a larger population.

## Conclusions

Anterior gingival enlargement does impact on OHRQoL and it is associated to sociodemographic characteristics in young subjects under orthodontic fixed treatment. Thus, it is possible that prevention and/or treatment of gingival enlargement may improve the OHRQoL of orthodontic patients. However, further studies are required to understand this issue better.

## Competing interests

The authors declare that they have no competing interests.

## Authors’ contributions

FBZ and CKR conceived of the study and designed the study; FBZ and TMA conducted the statistical Analyses; FBZ, CKR, RPA and TMPP drafted the manuscript. All authors read and approved the final manuscript.

## Pre-publication history

The pre-publication history for this paper can be accessed here:

http://www.biomedcentral.com/1472-6831/12/53/prepub
